# *VD9136* Positively Modulates the Pathogenicity of *Verticillium dahliae* to Cotton

**DOI:** 10.3390/ijms27083558

**Published:** 2026-04-16

**Authors:** Kailu Chen, Rui Tang, Qing Xu, Ziqi Li, Xuebin Wang, Shandang Shi, Fei Wang, Lingling Chen, Hongbin Li

**Affiliations:** Key Laboratory of Oasis Town and Mountain-Basin System Ecology of Xinjiang Production and Construction Corps, Key Laboratory of Xinjiang Phytomedicine Resource and Utilization of Ministry of Education, College of Life Sciences, Shihezi University, Shihezi 832003, China; ckl3224832883@163.com (K.C.); tr2604791944@163.com (R.T.); xq19901526963@163.com (Q.X.); liziqi@xjshzu.com (Z.L.); wxb18096810179@163.com (X.W.); shi_shandang@163.com (S.S.); feiw@shzu.edu.cn (F.W.)

**Keywords:** Verticillium wilt, *Verticillium dahliae*, HIT family, cotton pathogenicity

## Abstract

Histidine triad (HIT) family proteins contain a conserved histidine triad motif and play key roles in fungal metabolism and pathogenicity. This study focused on *VD9136*, a member of the HIT family in *Verticillium dahliae*, aiming to elucidate its biological function and mechanism underlying its role in cotton pathogenesis. A systematic investigation of the *VD9136* gene in *V. dahliae* was conducted using bioinformatics analysis, gene knockout, genetic complementation, and pathogenicity assays. The results showed that VD9136 protein consists of 136 amino acids and is a stable, neutral, and weakly hydrophilic protein that lacks transmembrane domains and signal peptides; it is localized to the extracellular space via a non-classical secretion pathway. Its secondary structure is predominantly composed of α-helices and random coils. Phylogenetic analysis revealed that *VD9136* is closely related to VliHIT, a homologous protein from *V. longisporum*, the pathogen responsible for Verticillium wilt in rapeseed. The promoter region of *VD9136* contains multiple cis-acting elements, including light-responsive, hormone-responsive, and stress-responsive elements, indicating that its transcription may be regulated by multiple signaling pathways. *VD9136* was significantly upregulated during the early stage of cotton infection (6–24 h post-inoculation). Pathogenicity assays demonstrated that *V. dahliae* knockout mutants lacking *VD9136* exhibited a significant reduction in virulence, as evidenced by a lower disease index, decreased fungal biomass within plant tissues, and attenuated vascular browning in cotton plants. The pathogenic phenotype was successfully restored in genetic complementation strains. This study identified *VD9136* as a key regulatory factor in the pathogenic process of *V. dahliae*, and its loss of function reduces the pathogenicity of *V. dahliae*. The findings provide a theoretical basis for elucidating the pathogenic mechanism of cotton Verticillium wilt and for developing corresponding prevention and control strategies.

## 1. Introduction

Cotton is a vital commercial crop worldwide and contributes significantly to the advancement of the textile sector and national economic development. Owing to its wide range of uses and large planting area, the stability of cotton production is closely linked to the sustainable development of the social economy [1]. Xinjiang is not only the main cotton-producing area but also the core yield–supply region in China, where the cotton industry has become an important pillar of local economic development [2]. However, the emergence of plant diseases and insect pests exerts a severe negative impact on cotton output, leading to yield reductions ranging from 15% to 50% [3]. Approximately forty types of cotton diseases have been reported in China to date. Among them, cotton Verticillium wilt induced by *Verticillium dahliae* is the most destructive fungal disease, which significantly reduces the yield and quality of cotton fibers and has become a key factor hindering the stable development of the cotton industry in Xinjiang [4]. *V. dahliae* is a globally distributed soil-borne vascular pathogenic fungus capable of infecting more than 600 plant species across 38 families, with particularly severe damage to economic crops such as cotton, tomato and potato [3,5]. The Verticillium wilt caused by this pathogen is referred to as “cotton cancer” due to the long dormancy period of its microsclerotia (surviving in soil for more than 14 years), strong drug resistance and complex pathogenic mechanisms, which has long constrained agricultural production [3,5,6]. After cotton is infected by *V. dahliae*, typical symptoms such as leaf yellowing, wilting, and browning of stem xylem often occur; infection at the flowering stage can lead to bud and boll abscission, and even plant death in severe cases [5,7,8]. Although existing studies have comprehensively elucidated the intricate molecular pathogenic mechanisms of *V. dahliae* in cotton, emphasizing that the identification and functional characterization of pathogenicity-related genes are central to deciphering the fungal infection process and developing effective disease control strategies [3], research on the pathogenicity of this fungus remains relatively limited. Therefore, it is urgent to systematically elucidate its key molecular regulatory mechanisms to support the development of effective prevention and control strategies.

*V. dahliae* contains HIT (histidine triad) family proteins, which are usually involved in cellular metabolism and pathogenicity-related processes. HIT family proteins contain a conserved H × H × H motif that can bind zinc ions in vitro, and this motif is also an important component of nucleotide-binding sites [9,10]. Taking *VdThit* from the HIT family as an example, its HIT domain mediates thiamine transport. Knockout of the HIT domain function leads to the blockage of fungal pyruvate metabolism and reduced accumulation of key intermediates such as acetyl-CoA, thereby restricting hyphal growth and conidial germination and reducing nutrient acquisition [11]. Based on their catalytic specificity, sequence composition, and structural similarity, HIT proteins can be divided into five branches: the Hint branch (consisting of adenosine 5′-monophosphoramidase), the Fhit branch (mainly composed of diadenosine polyphosphate hydrolase), the GalT branch (consisting of UDP-galactose:1-phosphate uridylyltransferase), the DcpS branch (specifically cleaving methylated mRNA transcripts) and the aprataxin branch (associated with DNA repair mechanisms) [9,12,13,14]. In Saccharomyces cerevisiae, reduced steady-state level of Hit1 protein lead to significant rRNA processing defects [15]. In Candida albicans, the CaHINT protein with a HINT crystal structure has been identified [16]. In addition, the HIT domain of HIT family proteins in plants is mainly involved in stress response and disease resistance defense processes [17,18,19]. For example, Zhang and colleagues discovered in *Arabidopsis thaliana* that FHIT, a member of the HIT protein family, functions as a factor responsible for restarting mRNA translation. This protein alleviates the inhibition of translation caused by upstream open reading frames (uORFs), helps restore the translation efficiency of the main open reading frame (mORF) in the *ctr1-10* mutant line, and further modulates the ethylene signal transduction pathway, as well as plant growth-related phenotypes [17]. In *A. thaliana*, the HIT family protein HIT4 participates in mediating chromatin relaxation at chromocenters triggered by high temperature and the release of transcriptional gene silencing (TGS). It also plays a role in the heat-dependent reactivation of transcriptionally silent genomic regions, a process that fulfills a crucial function during the acquisition of heat tolerance in plants [18]. In cotton, the HIT domain of HIT proteins can bind nucleotide signaling molecules related to stress response (e.g., cAMP) and participate in the signal transduction of abiotic stresses such as drought and high salt, thereby maintaining cellular metabolic homeostasis [19]. In addition, Wang et al. found that HIT1 is involved in the regulation of pollen tube elongation [20]. Currently, research on HIT family proteins in *V. dahliae* is still scarce. Given that HIT family proteins contain a conserved histidine triad motif and are essential for basic metabolic regulation and stress response processes, we infer that these elements may also play a critical role in sustaining the pathogenicity of *V. dahliae*. Therefore, exploring the functions of HIT family proteins in *V. dahliae* not only helps advance research on their pathogenic roles but also provides key theoretical support for the prevention and control of cotton Verticillium wilt, which has important theoretical and practical significance for the sustainable management of this disease.

Through systematic analysis of the transcriptome data of *V. dahliae*, a HIT family member *VD9136* was identified in the *V. dahliae* strain V592, and its encoded protein contains a typical conserved histidine triad motif of the fungal HIT family [9]. On this basis, we hypothesized that *VD9136* acts as a key regulatory gene involved in modulating the pathogenic processes of *V. dahliae* during cotton infection. However, the biological function of the *VD9136* gene in the disease induction process of *V. dahliae* remains unclear. In this study, we combined bioinformatics analysis of sequence characteristics, prediction and analysis of promoter cis-acting elements, and pathogenicity assays to explore the expression patterns and functional roles of *VD9136* during the entire process of *V. dahliae* infection in cotton. This study is expected to clarify the regulatory role of *VD9136* in the pathogenicity of *V. dahliae*, thereby laying a solid theoretical foundation for further deciphering the molecular pathogenesis of cotton Verticillium wilt and providing novel insights for the development of effective disease control strategies.

## 2. Results

### 2.1. Analysis of Physicochemical Properties of the VD9136 Protein

**Figure 1:**
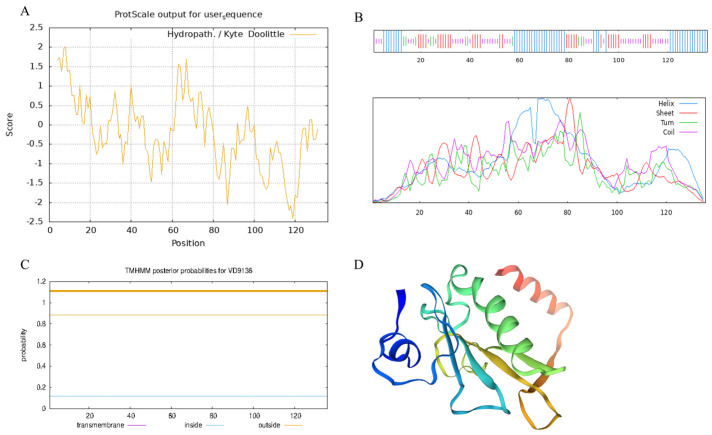
Physicochemical property analysis of the VD9136 protein. (**A**) Hydrophilicity analysis. (**B**) Transmembrane structure prediction. (**C**) Protein secondary structure analysis. (**D**) Tertiary structure prediction.

According to online analysis via ProtParam (ExPASy 3.0), the *VD9136* polypeptide comprises 136 amino acids, with a molecular mass of 15.31 kDa and a calculated pI of 7.09, indicating an overall neutral character. The aliphatic index was determined to be 97.50, while the grand average of hydropathicity (GRAVY) was calculated as −0.186, indicating weak hydrophilicity; its instability index was only 15.81, confirming that it is a stable protein. In summary, VD9136 is a stable, neutral protein with good thermal stability. The hydropathicity index (Kyte–Doolittle score) of the protein fluctuates between −2.5 and 2.0, confirming the overall weak hydrophilicity of the VD9136 protein (Figure 1A). TMHMM 2.0 prediction identified the VD9136 protein as a protein without transmembrane domains, and the entire polypeptide chain is localized in the extracellular region, indicating that the VD9136 protein may be a non-transmembrane, secreted, or extracellular protein (Figure 1C). SignalP prediction showed that the protein does not contain a typical signal peptide. In conclusion, the protein is a soluble stable protein without transmembrane regions that is released extracellularly through a non-classical secretion pathway. Protein secondary structure was predicted and analyzed using the SOPMA web server (INSERM Lyon, 2024), and its conformation is relatively rich in α-helices (36.03%) and random coils (33.09%), followed by β-sheets (22.79%) and β-turns (8.09%) with the lowest proportion, indicating that the protein has a stable structure and meets the functional requirements of the extracellular environment (Figure 1B). The tertiary structure was predicted using the SWISS-MODEL tool, which was consistent with the secondary structure prediction (Figure 1D).

### 2.2. Conservation and Evolutionary Analysis of the VD9136 Protein

**Figure 2:**
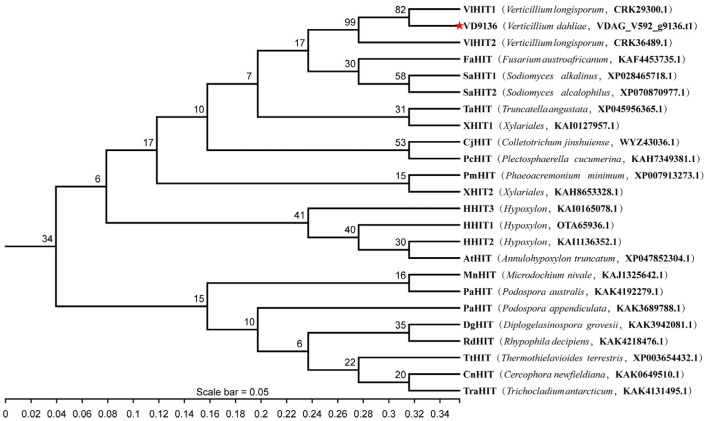
Evolutionary analysis of the VD9136 protein. Scale bar = 0.05 amino acid substitutions per site. VD9136 (*V. dahliae* V592) is highlighted with a red star.

**Figure 3:**
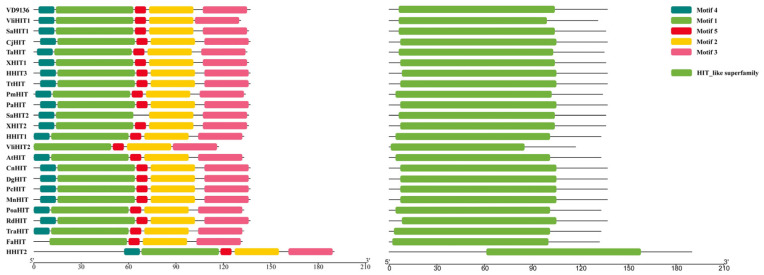
Analysis of conserved sequences and functional domain in the VD9136 protein.

In this study, 24 homologous protein sequences of VD9136 from 19 different species were collected using the BLASTP tool (BLAST + 2.17.0) in the NCBI database for phylogenetic analysis. Phylogenetic tree analysis results showed that *VD9136* from *V. dahliae* and VliHIT from *Verticillium longisporum* clustered into the same clade with a sequence similarity of 82%, indicating a close genetic relationship between them; these two proteins may originate from a common ancestor and maintain functional conservation during species evolution (Figure 2). Conserved motif and conserved domain analysis showed that all proteins contain the core HIT-like superfamily domain, and the composition and arrangement patterns of conserved motifs are highly consistent (Figure 3). These results were consistent with the sequence similarity analysis, collectively indicating that the HIT family-like proteins of *V. dahliae* have maintained structural and functional conservation during long-term evolution, providing a research basis for their similar roles in the pathogenic process of the strain.

### 2.3. Characterization of Cis-Acting Elements Within the VD9136 Gene Promoter Region

**Figure 4:**
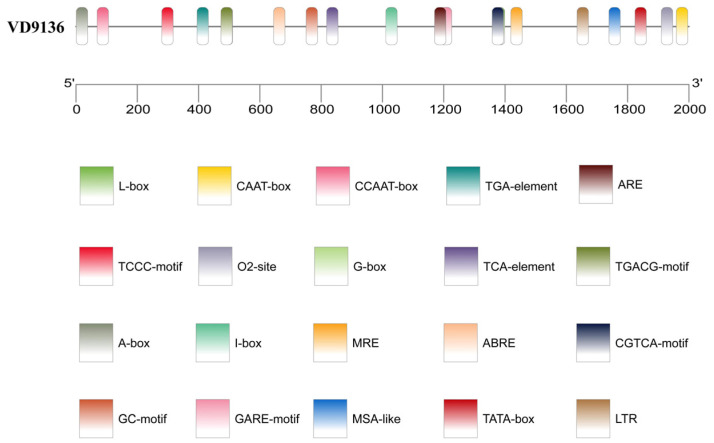
Cis-acting element prediction of the *VD9136* gene promoter.

Using the PlantCARE (Version 24.0) online analysis tool, we identified a variety of cis-acting elements in the 2000 bp promoter sequence upstream of the transcription start site of *VD9136*, including two core promoter elements (CAAT-box and TATA-box) and multiple functional response elements involved in light response (e.g., L-box, TCCC-motif, and I-box), hormone regulation (e.g., TGA-element, TGACG-motif, and CGTCA-motif), stress tolerance (e.g., ARE and LTR), secondary metabolism, and cell cycle regulation (e.g., O_2_-site and MSA-like element) (Figure 4). These findings suggest that the transcription of the *VD9136* gene can be modulated by various signaling pathways, including those related to light, hormones, and stress responses, providing molecular support for explaining its functions in physiological processes of *V. dahliae* such as sensing environmental signals, adapting to stress, and maintaining its own physiological homeostasis.

### 2.4. Expression Analysis of the VD9136 Gene During V. dahliae Infection

**Figure 5:**
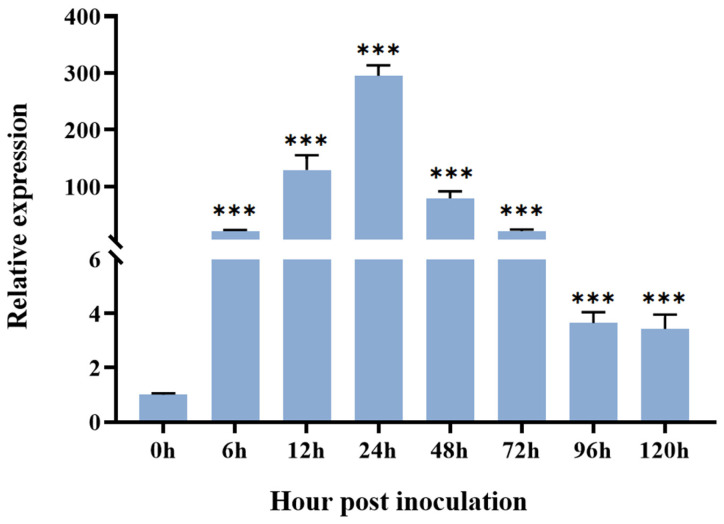
Expression Analysis of *VD9136* during *Verticillium dahliae* Infection in Cotton. *** indicates extremely significant difference (*p* < 0.001).

To explore the biological role of the *VD9136* gene during plant infection and pathogenesis in *V. dahliae*, the expression pattern of this gene during fungal infection was first analyzed. As shown in Figure 5, the transcription level of this gene exhibited an obvious upward trend at the initial phase of infection (6–24 h). The expression level was rapidly upregulated after inoculation and reached a peak at 24 h (approximately 300 times that at 0 h) and remained at a high level at the subsequent time points, still 3.5 times that at 0 h at 120 h. These results demonstrated that the *VD9136* gene was remarkably upregulated at the preliminary stage of host infection by the pathogen, suggesting that the *VD9136* gene may be involved in the pathogenic process of *V. dahliae*.

### 2.5. VD9136 Positively Regulates the Virulence of V. dahliae to Cotton

**Figure 6:**
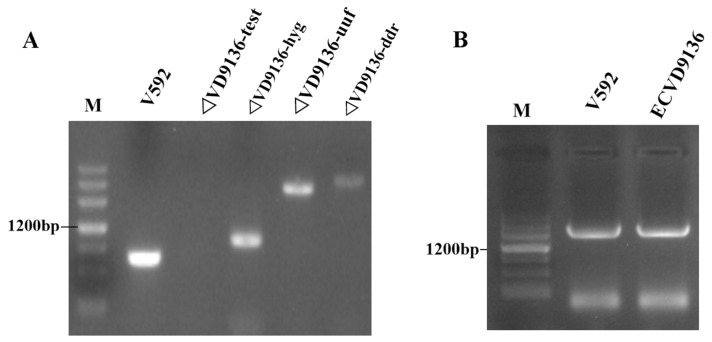
PCR identification of *VD9136* knockout and complementation mutants in *V. dahliae*. (**A**) PCR verification of knockout mutant. (**B**) PCR verification of complemented mutant. M, Marker III; V592, wild-type strain; ΔVD9136, knockout mutants; ECVD9136, complementation mutants; test, PCR detection using primers specific to the target gene; hyg, PCR detection using hygromycin resistance gene-specific primers; uuf, PCR detection using a forward primer at approximately 1100 bp upstream of the target gene combined with the hygromycin reverse primer; ddr, PCR detection using a reverse primer at approximately 1100 bp downstream of the target gene combined with the hygromycin forward primer.

To clarify the intrinsic relationship between the *VD9136* gene and the pathogenicity of *V. dahliae*, an *Agrobacterium*-mediated method was used to knock out the *VD9136* gene in *V. dahliae* to obtain knockout mutant strains of this gene (Figure 6A). In addition, *Agrobacterium*-mediated T-DNA random insertion technology was used to complement the endogenous promoter of the *VD9136* gene and the target gene into the knockout mutant strains, and positively complemented mutant strains of the *VD9136* gene were obtained after screening (Figure 6B).

**Figure 7:**
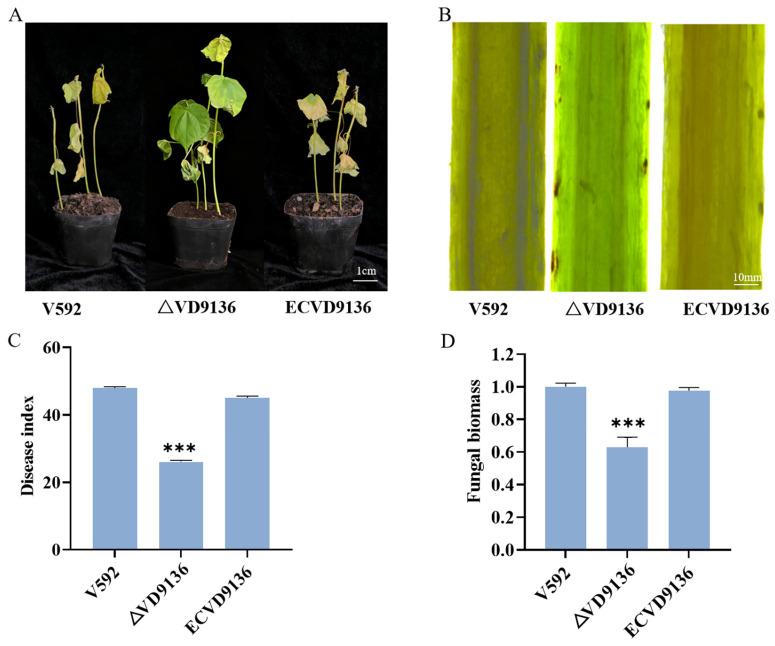
Pathogenicity analysis of *VD9136*. (**A**) Disease symptoms at 21 days post-inoculation (dpi) with wild-type and mutant strains. (**B**) Vascular bundle browning in cotton stem tissues. (**C**) Investigation of disease index at 21 dpi after inoculation with all strains. (**D**) Quantification of pathogen DNA in cotton stems at 21 days after inoculation. *Vdβt* served as the amplified marker gene, and the *Act* gene of Gossypium hirsutum was adopted as the internal reference. V592, wild-type strain; ΔVD9136, knockout mutants; ECVD9136, complementation mutants. *** indicates extremely significant difference (*p* < 0.001).

To clarify the function of the *VD9136* gene in the pathogenic process of *V. dahliae*, pathogenicity assays were carried out on the susceptible cotton variety Jimian 11 using three strains: wild-type (V592), knockout mutant (Δ*VD9136*), and complemented mutant (ECVD9136). Phenotypic observation revealed that cotton inoculated with the Δ*VD9136* strain showed mild wilting and yellowing symptoms, with symptoms significantly less severe than those inoculated with the wild-type and complemented strains (Figure 7A). At 21 days post-inoculation, the disease index (DI) of cotton plants infected by the knockout mutant was 26.05 ± 0.50, which was significantly lower than that of cotton plants infected by the wild-type (48.0 ± 0.30) and the complemented mutant (45.1 ± 0.50) (Figure 7C). Stem section results showed that the vascular browning degree of cotton infected by the knockout mutant strain was milder than that of cotton infected by the wild-type strain and the complemented mutant strain (Figure 7B). In addition, fungal biomass detection showed that the fungal biomass in cotton infected by the knockout mutant was lower than that in cotton infected by the wild-type and complemented mutant strains (Figure 7D). Collectively, these findings demonstrate that the *VD9136* gene contributes positively to the virulence of *V. dahliae*.

## 3. Discussion

The histidine triad (HIT) family genes are widely distributed across organisms, including prokaryotes, yeasts, and mammals, and they all harbor the characteristic conserved histidine triad motif (H × H × H) [9]. The catalytic activity of proteins in this family relies on a stable core domain, whereas their flexible regions facilitate binding to diverse substrate molecules [16]. In this study, the protein encoded by the *V. dahliae* HIT family gene *VD9136* was identified as a stable, neutral, and weakly hydrophilic protein with no transmembrane domain or classical signal peptide detected. These results indicate that the protein encoded by this gene does not function via the classical signal peptide-mediated secretory pathway or transmembrane transport. Instead, its characteristics are consistent with those of known virulence factors such as thioredoxin VdTrx1 [21] and extracellular superoxide dismutase VdSOD1 [22], which exert pathogenic functions by entering host intercellular spaces through non-classical secretion mechanisms. Moreover, the secretion of such effector proteins represents a major strategy employed by fungal and oomycete pathogens to infect host plants [23], suggesting that the *V. dahliae* VD9136 protein may be translocated extracellularly via non-classical secretion pathways and subsequently act rapidly at the host–pathogen interface. Phylogenetic analysis further revealed that the *VD9136* protein shares high sequence identity with the VliHIT protein from *Verticillium longisporum*, and the two cluster into the same evolutionary clade, indicating their close evolutionary relationship and conserved structural features. *Verticillium longisporum* is a vascular pathogen closely related to *V. dahliae* and causes Verticillium wilt in cruciferous plants [24]. Given this close phylogenetic relationship, the high conservation of the *VD9136* gene within the genus *Verticillium* further suggests that this gene performs conserved core physiological functions and may play a conserved role in the pathogenicity of *V. dahliae*.

During plant–pathogen interactions, cis-acting elements in gene promoter regions are key mediators of transcriptional regulation in response to environmental and host signals [25]. For example, the promoter of the *V. dahliae VdNoxB* gene contains oxidative stress-responsive elements that can be activated by reactive oxygen species signals, thereby regulating appressorium development and fungal pathogenicity [26]; in cotton, TGACG motifs and TCA elements in the promoters of defense genes can be recognized by flagellin FLiS, thereby activating salicylic acid and jasmonic acid signaling pathways and enhancing cotton resistance to Verticillium wilt [27]. In this study, multiple cis-acting elements, including light-responsive elements, hormone-responsive elements, and stress-responsive elements, were identified in the promoter region of the *VD9136* gene. The presence of these elements indicates that transcription of the *VD9136* gene is finely regulated by multiple signaling pathways, enabling *V. dahliae* to perceive host light conditions, hormone signals, and changes in stress conditions during infection. This sophisticated transcriptional regulatory pattern allows the strain to rapidly respond to changes in the host microenvironment, consistent with its temporal expression pattern during cotton infection. The results of this study showed that the transcription level of the *VD9136* gene was significantly upregulated at 24 h post-infection compared with the initial stage. This indicates that, under the regulation of promoter cis-acting elements, the *VD9136* gene is rapidly and strongly induced at the early stage of infection, laying a molecular foundation for *V. dahliae* to initiate pathogenicity-related programs and successfully colonize cotton.

Previous studies have confirmed that the *V. dahliae* HIT family gene *VdThit* encodes a thiamine transporter and is an essential gene for the pathogenicity of this fungus. Knockout of this gene impairs the pathogenicity of *V. dahliae* to cotton, resulting in significantly reduced disease index and fungal biomass in planta [11]. Similarly, knockout of the gene encoding the secreted protein VdCUE in *V. dahliae* also leads to a significant attenuation of strain virulence [28]. To verify the pathogenic function of the VD9136 protein, this study constructed *VD9136* knockout mutants and complemented mutants. Pathogenicity assays showed that, compared with the wild-type strain V592, cotton plants infected by *VD9136* knockout mutants exhibited a significantly lower disease index, significantly decreased fungal biomass in stem tissues, and markedly alleviated typical vascular browning symptoms of Verticillium wilt, which are highly consistent with the phenotypic characteristics after knockout of other virulence genes in *V. dahliae* [11,28]. Importantly, the pathogenic phenotype of the complemented mutants was largely restored to the wild-type level. These results confirm that the reduced virulence of the mutants is directly caused by the loss of *VD9136* function, and also indicate that *VD9136* positively regulates the pathogenicity of *V. dahliae* to cotton.

In summary, *VD9136* is a key pathogenic regulator in *V. dahliae*, and knockout of this gene significantly weakens the virulence of *V. dahliae* to cotton. Future studies can employ molecular interaction techniques such as yeast two-hybrid and co-immunoprecipitation to screen and identify host and fungal proteins interacting with *VD9136*, and clarify the regulatory network mediated by *VD9136* combined with transcriptomic and metabolomic analyses. The results of this study enrich our understanding of the pathogenic functions of HIT family proteins in *V. dahliae*, provide new clues for dissecting the molecular pathogenic mechanism of cotton Verticillium wilt, and also offer potential molecular targets for the control of this disease.

## 4. Materials and Methods

### 4.1. Experimental Materials

The cotton variety Jimian 11 was used as the plant material in this study. The highly virulent *V. dahliae* strain V592 and the p*MC-GFP* plasmid were preserved in our laboratory. The p*GKO-HPT* plasmid was kindly supplied by the Institute of Cotton Research, Chinese Academy of Agricultural Sciences (Zhengzhou, China). Plant Total RNA Extraction Kit (RC401-01), High-Fidelity Taq DNA Polymerase (P525-01), Plant DNA Extraction Kit (DC104-01), and Homologous Recombinase (C115) were purchased from Nanjing Vazyme Biotech Co., Ltd. (Nanjing, China). Cefotaxime (C8240), Hygromycin (H8080), and Kanamycin Sulfate (K8020) were purchased from Beijing Solarbio Science & Technology Co., Ltd. (Beijing, China). *Agrobacterium tumefaciens* EHA105 competent cells (AC1010) and *Escherichia coli* DH5α competent cells (DL1001S) were purchased from Shanghai Weidi Biotechnology Co., Ltd. (Shanghai, China). The primer synthesis and nucleic acid sequencing services required for this study were commissioned to Xinjiang Youkang Biotechnology Co., Ltd. (Urumqi, China).

### 4.2. Bioinformatics Analysis of the VD9136 Gene

The online platform ExPASy (Version 3.0) was used to analyze the basic physicochemical characteristics of the VD9136 protein, including amino acid sequence, isoelectric point, molecular weight, and hydrophobicity, and to predict its tertiary structure [29]. Transmembrane domains and signal peptides were predicted using the TMHMM 2.0 server and SignalP-6.0 server, respectively [30,31]. Protein secondary structure was predicted using the SOPMA (INSERM Lyon, 2024) server [32]. The phylogenetic tree was constructed using MEGA 11.0 software [33]. The maximum likelihood (ML) method was employed based on the JTT + G amino acid substitution model, and node support values were estimated using 1000 bootstrap replicates. The conserved motifs and conserved domains of the VD9136 protein were analyzed using the MEME (version 5.5.7) server and CD-Search tool (Version 3.18), respectively, and visualized using TBtools-II (Version 2.410) software [32,34,35]. The promoter sequence of the *VD9136* gene in the 2000 bp region upstream of the transcription start site was retrieved from the laboratory database and submitted to the PlantCARE (Version 24.0) server for cis-acting element prediction [36]. Finally, the distribution map was drawn using TBtools-II (Version 2.410) software [35]. Online analysis URLs used in this study are provided in Table S1.

### 4.3. Preparation of V. dahliae Conidial Suspension

The wild-type and mutant strains of *V. dahliae* were each inoculated onto potato dextrose agar (PDA) plates for activation. After 5 days, the strains were inoculated into Czapek liquid medium and cultured with shaking at 25 °C for 3 days at a constant speed. The mycelia were removed by filtration through eight layers of sterile gauze, and the filtrate was centrifuged at 5000 rpm for 5 min to collect the conidial pellets. The pellets were resuspended in double-distilled water, and the concentration of the conidial suspension was adjusted to 1 × 10^7^ cfu/mL for subsequent experiments [6,37].

### 4.4. Sample Collection of Cotton Infected by V. dahliae

When cotton seedlings had developed the second true leaf, the root-dipping method was used for inoculation [6]. Plants were uprooted from nutrient soil, and the soil particles attached to the roots were rinsed thoroughly with water. The entire root system of the plants was then immersed in the prepared conidial suspension for 30 min. After inoculation, the plants were removed and rinsed with sterile water. Cotton root samples were collected at 0 h, 6 h, 12 h, 24 h, 48 h, 72 h, 96 h, and 120 h post-inoculation for subsequent experiments.

### 4.5. Construction of the VD9136 Gene Knockout Vector of V. dahliae

Agrobacterium-mediated transformation technology was used for gene knockout [6], and the experimental procedures are detailed as follows: (1) Genomic DNA isolated from *V. dahliae* isolate V592 was used as the template for amplifying the 1 kb upstream and downstream regions flanking the *VD9136* locus. These two flanking DNA fragments were integrated into the linearized pGKO plasmid by means of homologous recombination, which was then transformed into *E. coli* competent cells DH5α. Candidate strains were then screened by agarose gel electrophoresis, and the correct knockout vector was obtained after sequencing confirmation. (2) Plasmids were extracted from the strains identified as positive by sequencing and the obtained plasmids were introduced into *Agrobacterium tumefaciens* EHA105, and positive clones were obtained using colony PCR. (3) Positive clones were harvested and adjusted to an optical density at 600 nm (OD_600_) of 0.6–0.8, then transferred to the pre-prepared conidial suspension of wild-type *V. dahliae* V592, mixed thoroughly, and spread onto Induction Medium (IM) agar plates supplemented with kanamycin sulfate, cefotaxime, and hygromycin. (4) Single colonies were selected for purification, followed by colony PCR. For colony PCR detection, three pairs of primers were designed and synthesized using Primer Premier 5.0 software (primers in Supplementary Table S2): the first pair used the upstream primer (UUF, approximately 100 bp outside the upstream fragment) and the downstream primer HPT-5R; the second pair used the upstream primer HPT-3F and the downstream primer (DDR, about 100 bp outside the downstream fragment); in the final PCR amplification step, the target gene-specific primers were used for amplification. After three rounds of amplification, consistent results with the expected outcomes indicated successful detection, and the strain was identified as a knockout mutant.

### 4.6. Construction of the VD9136 Gene Complementation Vector of V. dahliae

The endogenous promoter of the *VD9136* gene and the target gene were ligated into the linearized p*MC-GFP* vector by homologous recombination. The sequencing-verified recombinant vector was subsequently introduced into *Agrobacterium tumefaciens* EHA105 (AC1010, Weidi Bio, Shanghai, China) competent cells. PCR amplification was performed using the gene-specific upstream primer and the universal primer of the p*MC-GFP* vector. Other experimental steps for gene complementation were performed as described in Section 4.5.

### 4.7. Pathogenicity Identification of V. dahliae Mutant Strains

Cotton seedlings at the two-true-leaf stage were inoculated via the root-dipping method [6]. Root systems were thoroughly rinsed, soaked in the conidial suspension for 30 min, and then transplanted into fresh potting soil and incubated in a cotton growth chamber at 28 °C under a 16 h light/8 h dark photoperiod. Disease occurrence was investigated three weeks later, and disease severity was divided into different grades according to leaf symptoms: Grade 0 (healthy plants), Grade 1 (1–2 cotyledons with disease symptoms), Grade 2 (cotyledons and one true leaf infected), Grade 3 (both true leaves infected), and Grade 4 (all leaves infected). Disease severity was quantitatively assessed by calculating the disease index based on the graded disease incidence records using the formula shown below:

Disease index = ∑ (Number of diseased plants at each grade × Corresponding disease grade value)/(Total number of plants × Maximum disease grade value) × 100

### 4.8. Determination of Relative Fungal Biomass in Infected Plants

Stem segments from cotton plants inoculated with the fungal suspension were excised and rapidly frozen in liquid nitrogen. Total genomic DNA of the samples was extracted according to the protocol of the FastPure^®^ Plant DNA Isolation Mini Kit (DC104, Vazyme, Nanjing, China). Using the cotton Act gene as the internal reference and the *V. dahliae* Vdβt gene as the target, quantitative real-time PCR (qPCR) was performed. The 2^−ΔΔCT^ method was used to analyze the relative expression level of the target gene, and the expression level was used to indirectly characterize the relative fungal biomass in cotton stem tissues. Each sample was prepared with three independent biological replicates and three technical replicates [6]. Primers used are listed in Table 1.

### 4.9. Observation of Browning Phenotype in Xylem of Infected Plants

Twenty-one days after inoculation with *V. dahliae*, cotton plants with consistent growth phenotypes were selected. The surface of the tested stem segments was repeatedly wiped and disinfected with 75% (*v*/*v*) ethanol. Stem segments (1 cm in length) at the same node of different plants were excised with a sterile scalpel and were longitudinally split and placed under a Leica microscope to observe and record the browning status of vascular bundles in the xylem and the phenotypes were photographed for documentation [6].

## Figures and Tables

**Table 1:** Primers for qRT-PCR.

Primer Name	Primer Sequence (from 5′ to 3′)
*Vdβt*-F	AACAACAGTCCGATGGATAATTC
*Vdβt*-R	GTACCGGGCTCGAGATCG
*Gh-qPCR-Act-F*	CGGCTACCACATCCAAGGAA
*Gh-qPCR-Act-*R	TGTCACTACCTCCCCGTGTCA

## Data Availability

Data are contained within this article and the Supplementary Materials.

## Supplementary Materials

The following supporting information can be downloaded at: https://www.mdpi.com/article/10.3390/ijms27083558/s1.
